# Animal models to improve our understanding and treatment of suicidal behavior

**DOI:** 10.1038/tp.2017.50

**Published:** 2017-04-11

**Authors:** T D Gould, P Georgiou, L A Brenner, L Brundin, A Can, P Courtet, Z R Donaldson, Y Dwivedi, S Guillaume, I I Gottesman, S Kanekar, C A Lowry, P F Renshaw, D Rujescu, E G Smith, G Turecki, P Zanos, C A Zarate, P A Zunszain, T T Postolache

**Affiliations:** 1 Department of Psychiatry, University of Maryland School of Medicine, Baltimore, MD, USA; 2 Department of Pharmacology, University of Maryland School of Medicine, Baltimore, MD, USA; 3 Department of Anatomy and Neurobiology, University of Maryland School of Medicine, Baltimore, MD, USA; 4 Department of Psychiatry, University of Colorado Anschutz Medical Campus, Aurora, CO, USA; 5 Rocky Mountain Mental Illness Research Education and Clinical Center, Denver, CO, USA; 6 Military and Veteran Microbiome Consortium for Research and Education, U.S. Department of Veterans Affairs, Washington, DC, USA; 7 Department of Physical Medicine and Rehabilitation, University of Colorado Anschutz Medical Campus, Aurora, CO, USA; 8 Center for Neurodegenerative Science, Van Andel Research Institute, Grand Rapids, MI, USA; 9 Department of Psychology, Notre Dame of Maryland University, Baltimore, MD, USA; 10 Department of Emergency Psychiatry and Post Acute Care, CHU Montpellier, Montpellier, France; 11 Université Montpellier, Inserm U1061, Montpellier, France; 12 Department of Molecular, Cellular, and Developmental Biology, University of Colorado Boulder, Boulder, CO, USA; 13 Department of Psychology, University of Colorado, Boulder, Boulder, CO, USA; 14 Department of Neuroscience, University of Colorado Boulder, Boulder, CO, USA; 15 Department of Psychiatry and Behavioral Neurobiology, University of Alabama at Birmingham, Birmingham, AL, USA; 16 Department of Psychology, University of Minnesota, Minneapolis, MN, USA; 17 Department of Psychiatry, University of Minnesota Medical School, Minneapolis, MN, USA; 18 Department of Psychiatry, University of Utah, Salt Lake City, UT, USA; 19 Department of Integrative Physiology and Center for Neuroscience, University of Colorado Boulder, Boulder, CO, USA; 20 Department of Physical Medicine and Rehabilitation and Center for Neuroscience, University of Colorado Anschutz Medical Campus, Aurora, CO, USA; 21 Department of Psychiatry, University of Halle-Wittenberg, Halle, Germany; 22 Edith Nourse Rogers Memorial Veterans Hospital, Bedford, MA, USA; 23 Department of Psychiatry, McGill University, Montreal, QC, Canada; 24 Experimental Therapeutics and Pathophysiology Branch, Intramural Research Program, National Institute of Mental Health, National Institutes of Health, Bethesda, MD, USA; 25 Department of Psychological Medicine, Institute of Psychiatry, Psychology and Neuroscience, King's College London, London, UK; 26 VISN 5 Mental Illness Research Education and Clinical Center, Baltimore MD, USA

## Abstract

Worldwide, suicide is a leading cause of death. Although a sizable proportion of deaths by suicide may be preventable, it is well documented that despite major governmental and international investments in research, education and clinical practice suicide rates have not diminished and are even increasing among several at-risk populations. Although nonhuman animals do not engage in suicidal behavior amenable to translational studies, we argue that animal model systems are necessary to investigate candidate endophenotypes of suicidal behavior and the neurobiology underlying these endophenotypes. Animal models are similarly a critical resource to help delineate treatment targets and pharmacological means to improve our ability to manage the risk of suicide. In particular, certain pathophysiological pathways to suicidal behavior, including stress and hypothalamic–pituitary–adrenal axis dysfunction, neurotransmitter system abnormalities, endocrine and neuroimmune changes, aggression, impulsivity and decision-making deficits, as well as the role of critical interactions between genetic and epigenetic factors, development and environmental risk factors can be modeled in laboratory animals. We broadly describe human biological findings, as well as protective effects of medications such as lithium, clozapine, and ketamine associated with modifying risk of engaging in suicidal behavior that are readily translatable to animal models. Endophenotypes of suicidal behavior, studied in animal models, are further useful for moving observed associations with harmful environmental factors (for example, childhood adversity, mechanical trauma aeroallergens, pathogens, inflammation triggers) from association to causation, and developing preventative strategies. Further study in animals will contribute to a more informed, comprehensive, accelerated and ultimately impactful suicide research portfolio.

## Introduction

Suicide is the tenth leading cause of death in the United States, and the fifteenth worldwide, making it more lethal than common diseases like hypertension, liver disease or Parkinson's disease.^1^ For example, it has recently been documented that from 1999 to 2014, the suicide rate in the United States increased by 24%, with the greatest increase occurring more recently, after 2006.^2^ Although many deaths by suicide are likely preventable, suicide rates have been particularly resilient to multilevel interventions. In 2012, the rate of suicide among those serving in the United States uniformed services surpassed that of combat deaths in war zones.^3, 4^ Clearly, suicide prevention efforts demand increased attention. A major challenge is the broad conceptual gap between biological research in suicidal behavior and the current clinical practice of managing suicide risk.

Thus far, multiple research efforts in suicide prevention have been dedicated to psychological and socioeconomic factors leading to suicide, including better control of availability of lethal means. In addition, there is also evidence suggesting that some medications - lithium and clozapine - reduce the risk of suicidal behavior.^5^ Clinical risk (triggers) and protective (deterrents) factors have been extensively covered and summarized elsewhere.^6, 7, 8, 9, 10^ There are also extensive efforts directed at understanding the dysregulation of human brain functions^11^ and behavioral repertoire associated with ‘suicidal behavior', which refers to a heterogeneous outcome of suicide attempts and suicide completions.^12^

Support for the study of suicidal behavior as a scientific entity is multifold.^13^ Although it is well known that the nature of a suicidal act (suicide attempt or completed suicide) may be influenced by gender, age, availability of a lethal mean, and specific suicidal dimensions like suicidal intent and medical lethality, these behaviors seem to have more in common and are often distinguished from another component of the suicidal process, suicidal ideation. The clinical profiles of suicide attempters and completers significantly overlap.^14^ In addition, a previous suicide attempt is among the strongest predictors of future suicide.^15^ In contrast, suicidal ideation, (‘suicidality') often a major reason for hospitalization, is a significantly weaker predictor of suicide than attempts.

Epidemiological genetics studies indicate that suicide attempts and completed suicides may share a common genetic basis.^16^ Furthermore, in contrast to suicidal behavior, there is limited support that suicidal thoughts run in families, or that they are predictive of suicide attempts or completions within families.^17^ Finally, suicide attempts and suicide completions share many neurobiological correlates^18, 19^ and are thus commonly studied together. A historic turning point in suicidology resulted from the demonstration that psychobiological abnormalities are associated with vulnerability to suicidal behavior, independent of co-occurring psychiatric disorders.^18^

Even though suicidal behavior is the ultimate negative psychiatric outcome, the study of suicide can be difficult and experimental procedures among vulnerable populations can be ethically challenging. Although research at these levels of understanding is needed, current suicide rates suggest that additional approaches should also be strongly considered. Despite conceptual attempts to draw parallels between self-killing behavior in nonhuman animals and suicidal behavior in humans, our position is that suicide as it exists in humans is the outcome of a unique cognitive process. It involves insight, planning, and intent that is unlikely to be present in nonhuman animals, and in particular rodent model systems that are a focus of this review.^20, 21, 22^ Although we do not eliminate the possibility that nonhuman animals may engage in self-killing acts, such behavior is not the focus of this review. We argue that the increased use of animal model approaches with translational validity to study suicide neurobiology would allow the testing of hypotheses, as well as novel drug discovery leading to improved treatments for the prevention of suicide. Hypotheses tested first in animals and then in humans could point toward new treatments allowing a faster and more precise identification of potential molecular pathways and treatment targets, the avoidance of obstacles in human studies of suicide-related ethical concerns, as well as the issues with obtaining adequate sample sizes. Conversely, confirmation in animals of epidemiological and clinical observations in humans with suicidal behavior sublimated to simpler endophenotypes in thoroughly controlled experiments increases our capability to move associations closer toward causation, often unattainable in humans considering the degree of potential confounding, uncertain direction of causality, and interactions of multiple intertwined contributory factors. The authors on this review include both clinical and preclinical researchers, who have a shared interest in advancing the use of preclinical animal model approaches for the study of suicidal behavior.

## Animal models and suicide

Terms such as ‘animal model' or ‘model animals' or ‘animal assay' and ‘suicide' are not typically used in the same sentence. Although there are obvious limitations in assessing suicidal intent in animals, such as reproducing conceptualization, motivation, conscious planning, and the ultimate action of killing oneself,^22^ many components implicated in the neurobiology of suicidal behaviors, and the neurobiology of circuits delineated as relevant to ideation in humans, may be studied in animal models. Indeed, most animal ‘models' are only intended to recapitulate some aspects of human diseases, or components of neurobiology implicated in human psychiatric disorders, rather than the disorder itself. This approach limits ‘anthropomorphizing' by keeping the focus on neurobiological and pharmacological quantitative aspects that can be translated from humans to animal studies, which commonly use rodents. For example, in animal models of other uniquely human psychiatric conditions such as schizophrenia or depression, experimental animals cannot be labeled as psychotic or depressed. However, approaches that involve the use of model systems are still invaluable in understanding distinct component processes relevant to the disorder, are extensively used in the development of treatment approaches, and are applied toward identifying genetic and environmental influences for such conditions.^23^

**Figure 1:**
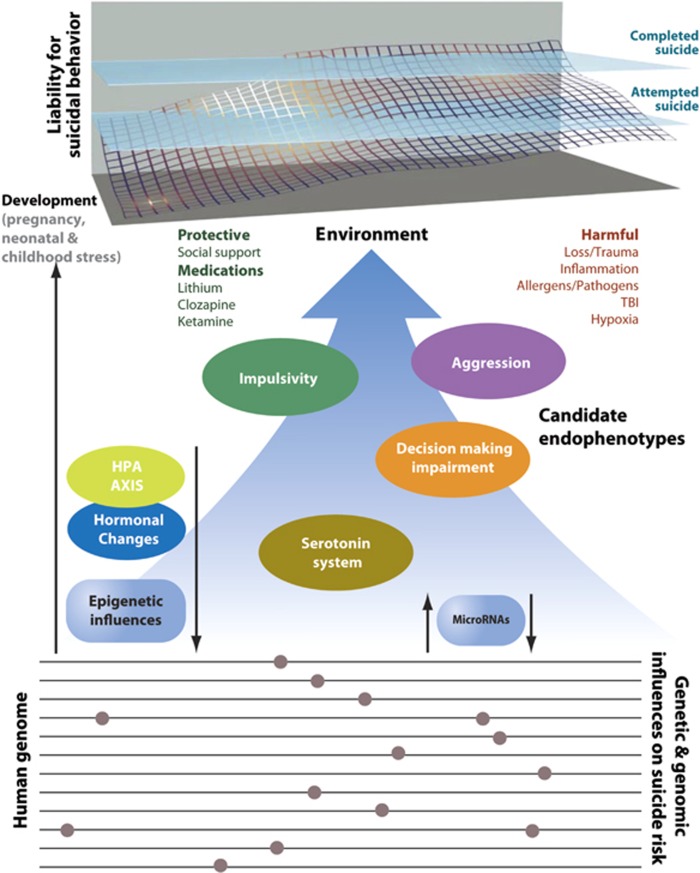
Heuristic model displaying candidate genes, endophenotypes and environmental risk factors implicated in suicidal behavior that may lend themselves to further study in animal model systems. The upper portion of the figure indicates the dynamic interplay among genetic, epigenetic and environmental factors that produce cumulative liability to complex behaviors such as suicide.^451^ Although attempted (non-successful) suicide does not always predate suicide as suggested on the reaction surface, it is a significant risk factor.^15^ None of the sections of this figure are meant to be definitive: gene loci, genes, candidate endophenotypes and links among these factors remain to be discovered, as well as factors that have not been fully evaluated. Environment, protective and harmful, includes a substantial number of sociological events unmentioned here because of our focus on the genetic and neurobiological correlates that may be modeled in nonhuman animals. Similarly, specific gene loci and genes were not included because of the current limitations in knowledge. TBI, traumatic brain injury.

A critically important aspect of developing an animal model approach to the study of suicidal behavior is to be realistic regarding what a particular model is measuring. The credibility of specific animal models as they pertain to human mental conditions are commonly evaluated as to whether they demonstrate face, construct, and predictive validity.^24, 25^ Face validity refers to phenotypes that contain similarities to humans who have the condition. Construct validity refers to processes that result in human pathology and are recapitulated with the model. Predictive validity relates to the capacity of a model to make predictions about the human condition. A model with predictive validity is sensitive to pharmacological and non-pharmacological interventions that effectively modify the condition in humans. We do not propose the development of animal models of suicidal behavior with face validity. However, predictive- or construct-based approaches can be formulated in the study of suicidal behavior, generally using suicide risk factors, and specifically candidate endophenotypes (quantifiable measures of neurobiological function) associated with suicide. Using this strategy, it becomes possible to translate clinical findings related to complex human behavior to animals and vice versa (Figure 1).^26^ We note that it is humans, not nonhuman animals, who manifest suicidal behavior. Basic studies in model animals can be used to understand fundamental mechanisms that underlie biological phenomena, but the biology underlying pathological dysregulation requires validation in humans.^27^

An example for such an endophenotype approach can be taken from schizophrenia research. Endophenotypes of schizophrenia, such as deficits in prepulse inhibition and impaired working memory performance, have been successfully translated to rodents to assist in understanding the neurobiology underlying the disorder, assessing the function of risk alleles, and developing novel therapeutic approaches.^28, 29, 30^ Such an approach could be used to more extensively study processes and behavior associated with suicide, whereby endophenotypes such as neurobiological, endocrine, neuroanatomical, and cognitive measures associated with suicidal behavior could be translatable to model animals (Figure 1).^31, 32, 33, 34, 35^

Suicide risk factors, such as stress and hypothalamic–pituitary–adrenal (HPA) axis dysfunction, hormonal and neurotransmitter system abnormalities, and endophenotypes including aggression, impulsivity, and decision-making deficits, can be modeled in laboratory animals. Animal models also present opportunities to study interactions between development and environment on critical neuronal circuits using manipulations not possible in humans. It is logistically and ethically more acceptable to investigate animal models than to perform human experimentation. For instance, moderators and modulators that have been associated with suicidal behavior can be investigated in relationship to endophenotypes in animal models without the risk to human life. Here, we briefly review some of these human biological findings. What is presented is not meant to be all inclusive, but rather to provide salient examples of approaches using rodent models that can be used to advance research designed to improve our understanding of the neurobiology and treatment of suicidal behavior.

## Molecular and hormonal pathways

We briefly present certain molecular pathways implicated in suicidal behavior that have been and can be studied readily in animal models.

### Genetics

The genetic risk of suicidal behavior is supported by family, twin, and adoption studies indicating that the tendency to commit suicidal acts has a genetic contribution that is independent of the heritability of psychopathology.^36^ One of the largest epidemiological studies, conducted by Mittendorfer-Rutz *et al*.,^37^ including 14 440 suicide attempters and 144 440 healthy controls, showed that the risk for suicide attempts increased when either parent died by suicide. Of course, early familial exposure to psychopathology and a suicidal attempt can have a detrimental effect on the family members, especially on those in a stage of emotional cognitive development such as young children and adolescents, and such effect would be unrelated to the genetic component. Addressing this issue, twin studies strongly suggested genetic contributions to liability for suicidal behavior with a heritability between 45 and 55%.^38^ Concordance for suicidal behavior was significantly more frequent among monozygotic (24.1%) than dizygotic twin pairs (2.3%). However, we cannot entirely discount that monozygotic twins experience an intricate unique psychological and emotional symbiosis throughout life, resulting in an increased potential of contagion of suicidal behavior that is not present in dizygotic twins. Adoption studies have also supported the role of genetic risks.^39, 40, 41^ For instance, suicide and indicators of severe psychiatric disorders in the biological parents were found to be similarly related to suicide in non-adopted and adopted children.^42^

There have been many single gene association studies undertaken. Many of these have focused on genes involved in serotonergic neurotransmission as this is crucial for the regulation of impulsive and aggressive behavior, which highly correlates with suicidal behavior.^18^ Recent years have brought an increase in genome-wide association studies, where greater than one million single-nucleotide polymorphisms can be studied. The first exome or whole-genome sequencing studies are underway. The full results of these studies are eagerly anticipated, and are expected to provide important new leads to study the neurobiology of suicidal behavior.

### Epigenetics

There is increasing evidence that environmental cues affect behavior through modifying gene expression; this includes epigenetic processes, which are lasting modifications of gene expression that do not change the coding sequence of the gene. Epigenetic modifications include mechanisms such as DNA methylation, histone modifications, as well as the effects of noncoding RNAs, which have emerged as the key molecular mechanisms that mediate brain plasticity in response to environmental changes.^43^

Maladaptive coping to the environment is a contributing factor to suicidal behavior in different age groups.^44, 45^ Cohort studies have provided data supporting predictive associations between abuse during early childhood, and psychopathology in adulthood, including increased anxiety, impulsivity, aggression, and increased risk of suicide or suicidal behavior.^46, 47^ Early-life adversity, including severe neglect, psychological, physical, or sexual abuse during childhood, has been consistently identified as a factor contributing to depression, substance abuse, and suicidality.^48, 49, 50, 51, 52, 53, 54, 55, 56, 57^ Several studies have reported an association of childhood abuse with earlier age of onset of psychopathology, a more severe course of illness, and poorer patient outcomes, including up to a 12-fold higher risk of engaging in suicidal behavior.^55, 58, 59, 60, 61, 62, 63, 64^ Evidence points to specific genes, especially those involved in the response to stress, as being epigenetically modified in response to early-life adversity.^65, 66, 67, 68, 69, 70, 71^ In addition, there is a growing literature implicating differential regulation of genes coding for neurotrophic, astrocytic, and neuroinflammatory proteins by epigenetic mechanisms in suicidal behavior.^72, 73, 74^ Epigenetic changes could therefore be the key mediators of the long-term effects of early-life adversity on behavior, psychopathology, and suicide risk.

Epigenetic modifications are possibly heritable, which may explain in part the familial aggregation of suicidal behavior, as well as of endophenotypes associated with such behavior. Further work exploring the inherited components of epigenetic regulation of suicide-related genes may explain the inherited vulnerability to mental illness and suicidal behavior in families with heavy loading of psychiatric conditions.^17, 75^

### MicroRNAs

MicroRNAs (miRNAs), a class of noncoding RNAs, have recently gained prominent attention for their role in neural plasticity and higher brain functioning.^76, 77, 78, 79^ These miRNAs bind to short sequences located predominantly within the 3′-untranslated region of messenger RNAs (mRNAs), and interfere with translation or stability of mRNAs with the potential to modulate disease phenotypes.^80^ Several miRNAs have been shown to regulate genes that are crucial in the neurobiology of suicidal behavior. For example, early childhood stress is significantly associated with upregulation of miR-16 and subsequent downregulation of the brain-derived neurotrophic factor (*BDNF*) gene in hippocampus.^81^ A strong miRNA-target interaction between miR-135 and the sodium-dependent serotonin (5-hydroxytryptamine; 5-HT) transporter and 5-HT receptor-1A transcripts has also been reported.^82^ Intriguingly, miR-135a levels are upregulated after the administration of antidepressants.^82^ Moreover, a decrease in 5-HT_1A_ receptors was observed in postmortem brain samples from depressed suicides compared with healthy controls.^83^ Interestingly, Jensen *et al*.^84^ reported that the expression of 5-HT_1B_ receptors, involved in aggressive behavior in humans, a critical endophenotype of suicide, is repressed by miR-96, which depends on the A-element of the 5-HT_1B_ receptor mRNA. Individuals who were homozygous for the ancestral A-allele had more conduct disorder behaviors than individuals with the G-allele.^84^ Another study found an association of a functional polymorphism in miR-124-1, which targets genes such as *BDNF* and *DRDF*, and aggressiveness, which could explain the effect of this miRNA on aggressive behavior.^85^ Recently, a gene-based association study showed potential involvement of DICER1 in suicide pathogenesis.^86^ DICER1 has a critical role in miRNA biosynthesis. Regulatory roles of miRNAs in polyamine gene expression in the prefrontal cortex of depressed suicide individuals have also been demonstrated.^87^ More recently, differential expression of miRNAs in the prefrontal cortex of patients with bipolar disorder, depression, and schizophrenia was identified; however, when suicides were separated from individuals who died from other causes, a subset of miRNAs were distinctly dysregulated in suicide subjects independent of psychiatric diagnosis.^88^ This raises an interesting possibility that there are miRNAs that may be involved in the regulation of brain circuits underlying behaviors associated with suicide across diagnostic boundaries.

Dwivedi and colleagues examined miRNA expression in the frontal cortex of rats that developed hopeless behavior (learned helplessness (LH)), a risk factor for suicidal behavior in humans, and those who did not develop such a behavior (non-learned helplessness (NLH)), even though they received similar shock paradigms.^89, 90^ They found that NLH rats showed a robust adaptive miRNA response to shocks, whereas LH rats showed a markedly blunted miRNA response. In addition, a large core co-expression module was identified, consisting of miRNAs that are strongly correlated with each other across individuals of the LH group but not with the NLH or the tested control group. The presence of such a module implies that the normal homeostatic miRNA response to shock is not merely absent or blunted in LH rats; rather, gene expression networks are actively reorganized in LH rats, which may give rise to a distinctive persistent phenotype. It will be critical, in the future, to identify the role of miRNA in regulating gene expression following stressful life events and modulation of circuits essential for the development and maintenance of endophenotypes associated with suicidal behavior.

### Serotonin system

Evidence suggests that the underlying neurobiology of suicidal behavior involves abnormalities in the 5-HT system, with findings supporting a downregulation of 5-HT function.^18, 83, 91, 92, 93, 94^ For example, cerebrospinal fluid (CSF) 5-hydroxyindoleacetic acid (5-HIAA, the major metabolite of 5-HT) has been shown to be lower among individuals who attempt and die by suicide than in controls, independent of other psychiatric diagnoses such as depression and schizophrenia.^95, 96, 97^ Low CSF 5-HIAA also predicts future suicide attempts and suicide.^95, 96, 97^ Irrespective of a psychiatric diagnosis, low CSF 5-HIAA levels are associated with aggression.^95, 98^ An association has also been identified between low CSF 5-HIAA levels and impulsivity, as identified by the performance on a human delayed reward discounting task.^99, 100, 101^ Similarly, dysregulation of the 5-HT system, in particular decreased 5-HT neurotransmission, has been associated with aggression and impulsivity in humans.^102^ Likewise, a number of preclinical studies in rodents implicate altered 5-HT neurotransmission with aggression. Similar to human 5-HIAA findings, both 5-HT and 5-HIAA levels, as well as 5-HT turnover, are negatively correlated with levels of aggression in rodents.^103, 104^ Administration of the tryptophan hydroxylase inhibitor para-chlorophenylalanine has been reported to increase aggression in both mice and rats.^105, 106^ Pharmacological studies have implicated 5-HT in control of impulsivity in the contexts of decreased latency to attack,^106, 107^ the five-choice serial reaction time test, and other tests of motor impulsivity,^108^ and some, but not all studies, indicate 5-HT-induced changes in tests of cognitive impulsivity.^108, 109, 110, 111, 112^ Serotonergic modulation of impulsive aggression implicated in suicide vulnerability can be studied in animal model systems in a straightforward manner within a context relevant to suicidal behavior.

The 5-HT system is hallmarked by 14 receptors with overlapping pharmacological properties. As such, genetic manipulation techniques have proven invaluable for determining which receptor populations modulate aggression and impulsivity. For instance, the *Htr1b* null mice exhibit increased aggression and impulsivity.^113, 114^ Using conditional knockout/knockdown approaches, which selectively delete specific genes in a tissue or brain region of scientific interest,^115^ recent work indicates that distinct neural circuits influence these phenotypes. The forebrain 5-HT_1B_ heteroreceptors determine adult aggressive behavior during development, while adult expression of a different population of 5-HT_1B_ heteroreceptors modulates impulsive behavior.^116^ Such a dissociation of the neural circuits underlying these two suicide-relevant endophenotypes may have important implications for prevention strategies and pharmacotherapies targeting the 5-HT_1B_ receptor system.^116^

Animal models may also help to clarify the findings regarding gene associations in the 5-HT system, and suicide-related endophenotypes. Associations have been studied for various serotonergic genes, including tryptophan hydroxylase genes: *TPH1*; *TPH2*, encoding the rate-limiting enzyme in the biosynthesis of 5-HT, 5-HT transporter gene: specifically, the 5-HT transporter-linked polymorphic region in *SLC6A4*, 5-HT receptor genes: *HTR1A*, *HTR2A*, *HTR1B*, *HTR2C* and monoamine oxidase A and B genes: *MAOA*, *MAOB*. In general, the results are inconsistent, making it difficult to draw reliable conclusions. Likewise, 5-HT receptor meta-analyses provide a heterogeneous picture, but in some cases, animal research can help clarify the putative role of a particular polymorphism.^117^ For instance, a *HTR2B* premature stop codon identified in the Finnish population is associated with severe impulsivity. Similarly, *Htr2b* knockout mice exhibit increased impulsivity and enhanced locomotor activity.^118^ Likewise, much work has been done *in vitro* to elucidate the putative functional mechanism underlying a common single-nucleotide polymorphism, rs6295, in the promoter of the *HTR1A* gene, which has been inconsistently associated with increased suicide risk.^119, 120^ These studies suggest that the G-allele of rs6295 may increase 5-HT_1A_ autoreceptor expression in serotonergic neurons. Mouse models with selectively altered 5-HT_1A_ autoreceptor levels have demonstrated a role for these receptors in modulating stress-coping behaviors.^121, 122^ Although these studies do not demonstrate a direct, functional role for rs6295, they support the idea that naturally occurring variation in 5-HT_1A_ receptor levels within the human population, such as those potentially attributable to rs6295, may be behaviorally relevant.

### Tryptophan degradation and the kynurenine pathway

The essential amino acid tryptophan is processed by the enzyme tryptophan hydroxylase to form serotonin. However, the largest part of tryptophan is broken down by the kynurenine pathway to form the kynurenine metabolites and the end product nicotinamide adenosine dinucleotide. A plausible mechanism that has been suggested to contribute to depressive symptoms is a relative deficiency in serotonin, caused by an increased amount of tryptophan shunted through the kynurenine pathway, instead of being utilized for serotonin production.^123^ Although experimental tryptophan depletion, as the result of experimental intake of a specific diet enriched in certain amino acids and depleted of tryptophan, can acutely suspend therapeutic control of depressive symptoms and induce dysphoria, anhedonia, and hopelessness, it has not been confirmed that such a deficiency state can occur as part of any clinical condition or that it modulates suicidal ideation or behavior.

It is now considered that dysregulated production of several neuroactive compounds through the kynurenine pathway can affect emotion and behavior. Inflammatory cytokines are potent inducers of this enzymatic pathway. Although it is important to point out that also, under non-inflammatory conditions, this pathway is active and breaking down over 90% of dietary tryptophan. Dietary and metabolic factors, such as glucose levels and obesity, as well as hormones and inflammation, all contribute to the regulation of the pathway, although the detailed mechanisms still remain to be determined.^124, 125^

Kynurenic acid and quinolinic acid (QUIN) are two metabolites produced by this pathway with multiple effects on neuroinflammation and glutamate neurotransmission in particular. QUIN is an *N*-methyl-d-aspartate (NMDA)-receptor (NMDAR) agonist, activating receptors containing the NR1+NR2A and the NR1+NR2B subunits.^126, 127^ On the other hand, kynurenic acid blocks several receptors, including the glycine site and the glutamate-recognition site of the NMDAR.^127^ Levels of QUIN are almost three times higher in CSF of suicide attempters than in healthy controls, correlating significantly with the levels of interleukin-6 (IL-6).^128^ This indicates that the kynurenine pathway is induced in the central nervous system of suicidal patients, presumably by an ongoing process of inflammation, with resultant downstream changes in glutamate neurotransmission. The imbalance of the kynurenine metabolites is evident in patients prone to suicidal behavior and may indicate a potential inherent vulnerability to stress and inflammation.^129^ Suicide attempters show elevated plasma kynurenine levels compared with non-suicidal depressive patients, who have levels of kynurenine similar to controls with no history of depression.^130^ There is no apparent activation of the kynurenine pathway, at least in the peripheral blood, of depressed patients without current suicidal thoughts.^131^ Consistent with these observations, Steiner *et al*.^132^ showed increased expression of QUIN-reactive microglia cells in the brains of depressed patients who died by suicide.

A recent report identified that an enzyme in the kynurenine pathway, the amino-β-carboxymuconate-semialdehyde-decarboxylase (ACMSD), may govern vulnerability to neuroinflammation through limiting the formation of the neurotoxic QUIN with production of the neuroprotective picolinic acid.^133^ Suicide attempters had trait-like decreased picolinic acid and picolinic acid/QUIN ratios in both blood and the CSF, suggesting a reduced activity of ACMSD. Furthermore, increased QUIN in the central nervous system was associated with a genetic variant in the *ACMSD* gene (C allele of the ACMSD single nucleotide polymorphism rs2121337; more prevalent in attempters than in controls). In conclusion, the ultimate fate of inflammation may depend on the ultimate neuroprotective versus excitotoxic outcome dictated by the (genetically determined) level of activity of downstream enzymes on the kynurenine pathway, such as ACMSD. In some individuals, the ACMSD activity may be high enough to neutralize, and even reverse, excitotoxic consequences of inflammation. In contrast, certain individuals, such as some among those who attempt suicide, may have limited ACMSD activity, with a cascading excitotoxic outcome in inflammation via the kynurenine pathway. This may explain some of the inconsistent findings across individual patients. ACMSD activity could be modeled in genetically modified rodents to evaluate its effects on endophenotypes of suicidal behavior, and screen for potential beneficial reduction of endophenotypic measures with ACMSD-targeting medications.

### Inflammation

Accumulating evidence indicates that inflammatory mediators are increased both in the central nervous system and in the periphery of patients with a history of suicidal behavior. Tonelli *et al*.^134^ found that mRNA transcription for the cytokines IL-4 and IL-13 was increased in the orbitofrontal cortex (OFC) of individuals who died by suicide. A second postmortem study published in the same year found pronounced microgliosis in the brains of patients who died from suicide.^135^ Recently, increased levels of IL-1β, IL-6 and tumor necrosis factor (TNF) at both the mRNA and protein levels were observed in the anterior prefrontal cortex of teenage suicide victims.^136^ Supporting the findings of increased inflammation in the central nervous system of suicidal patients, the cytokine IL-6 is significantly increased in the CSF of patients who attempt suicide compared with healthy controls.^137^

Suicidal behavior is also accompanied by changes in peripheral cytokine levels. An early study (1993) revealed that levels of soluble IL-2 receptor (IL-2R) are elevated in blood samples from suicide attempters.^138^ The plasma levels of cytokines IL-6 and TNF are increased, and those of IL-2 are decreased, in suicide attempters compared with both non-suicidal depressed patients and healthy controls.^139^ A composite inflammatory index, consisting of the sum of *z*-scores for C-reactive protein, IL-6, IL-10, and TNF, is associated with the occurrence of suicidal ideation, independent of both the severity of depression and whether the patients recently attempted suicide.^140^ Meta-analyses on this topic concluded that there are indeed aberrant cytokine levels in blood, CSF, and postmortem brain samples of patients who died by suicide.^141, 142, 143^ Blood levels of IL-1β and IL-6 appear to be most robustly associated with suicidal behavior^143^ among many markers of inflammation. In support of these meta-analyses, a recent gene-expression study showed that biological mechanisms related to stress, inflammation, and apoptosis may underlie, at least partly, suicidality and suicidal behavior.^144^ As a consequence, it has been suggested that suicide-risk assessment in the clinics might be improved by measuring peripheral inflammatory markers. Related biomarkers that have been associated with suicidality are blood levels of S100 calcium-binding protein B (S100B) and C-reactive protein. Falcone *et al*.^145^ reported that serum levels of S100B, a marker of inflammation in the central nervous system, were related to the intensity of suicidal ideation in teenagers with depression and psychosis.

The causal relationship between inflammation and depressive- and depressive-like symptoms has been confirmed in multiple animal models,^146, 147, 148, 149, 150, 151^ as well as in studies of healthy human volunteers who received injections of endotoxin, lipopolysaccharide, to induce inflammation.^152, 153^ Moreover, up to half of cancer and hepatitis C patients who receive interferon-α or IL-2 treatments develop depression and suicidality.^154, 155, 156, 157, 158^ Mechanistically, inflammation can affect the brain in several ways to modulate emotion and behavior. Specific cytokines are able to bind to specific neuronal receptors and thereby regulate neurotransmission. Endophenotypes associated with suicidal behavior, such as aggression, have been exacerbated (at least partially) by the effects of individual cytokines. For instance, IL-1β injected into the medial hypothalamus or dorsal periaqueductal gray acts on IL-1 type I receptors (IL-1RI) and 5-HT_2_ receptors to potentiate aggression in a feline model.^146, 149, 159^ An alternative pathway was uncovered by injecting IL-2 into the midbrain periaqueductal gray, which increases aggression through neurokinin NK (1) receptors.^147^ Moreover, TNF also appears to induce aggression. Mice lacking the TNF receptor do not exhibit aggressive behavior in the resident-intruder test.^150^ Individual cytokines might have very specific effects in the brain, and much still remains to be explored. In addition, as mentioned in earlier sections, inflammatory factors are modulators of the activity of enzymes in the kynurenine pathway producing neuroactive metabolites with effects on glutamate receptors. Finally, inflammation in the brain changes the turnover and metabolism of monoamines, including dopamine and 5-HT, and can thus have profound effects on several neurotransmitter systems. Importantly, several anti-inflammatory and kynurenine pathway-targeting medications are available and are of high interest for experimental testing in animals and then in patients with suicidal depression, based on the above observations. These medications may include anti-inflammatory agents, minocycline (through decreased microglia activation), glycogen synthase kinase-3 inhibitors (expected to reduce production of proinflammatory cytokines and aggressive behaviors), and infliximab (a monoclonal antibody against TNF).^160^ Although many are already United States FDA approved for other indications, these medications should first undergo a battery of testing in animals establishing their potential for improving depressive-like symptoms, aggression, impulsivity, and decision-making, to select the most suitable drugs for subsequent clinical trials in psychiatric patients.

### HPA axis

Dysregulation of HPA axis activity, as measured by the dexamethasone suppression test, has been associated with higher risk of suicide in patients with major depression.^161, 162^ Specifically, there is a reduction in the physiological responses downregulating cortisol levels following administration of the exogenous glucocorticoid dexamethasone. Evidence shows that, in humans who have experienced early-life adversity, responses to stress are altered^163, 164^ with increased corticotropin-releasing hormone levels.^165, 166^

Studies conducted in rodents and nonhuman primates have also shown that experiences in early-life have a long-term impact on the HPA axis.^167, 168, 169, 170^ Numerous studies conducted using a rat model of maternal care have demonstrated that the level of attention and care given to rat pups by dams is positively correlated with the hippocampal expression of glucocorticoid receptor (GR), which is a negative regulator of corticotropin-releasing hormone expression in the hypothalamus and is downstream of glucocorticoid-linked responses to stress.^170, 171, 172^ Importantly, attenuation of the response to stressful stimuli in this model is linked to epigenetic modifications of the GR gene where increased maternal licking/grooming is associated with decreased methylation of the GR promoter exon 1_7_ and increased expression of GR in the hippocampus.^170^ This finding has been partly reproduced in other models of early-life stress in rodents.^68^ Reports of changes in GR methylation in rats having had less favorable early-life experiences led to investigations of such potential mechanisms in human tissues.

Studies conducted on postmortem brain samples from individuals who had died by suicide showed that those who had a history of childhood abuse exhibited altered DNA methylation patterns on the GR gene in the hippocampus.^65, 70^ Evidence of such epigenetic dysregulation of the HPA axis provides an indication of ways in which early-life adversity may heighten the risk of suicide. The production and regulation of cortisol through this pathway is a crucial component of the organism's response to environmental cues. Dysregulation of cortisol can cause maladaptive coping behaviors; additionally, disruption of HPA activity is associated with suicide risk.^161^ Overall, early-life stress may result in dysregulation of the HPA axis and stress-coping behaviors, which in turn may increase the vulnerability to suicide.

### Gonadal hormones

Suicidal behavior has marked gender differences, including higher rates of attempts in females and higher rates of death by suicide in males in most countries, with China being a notable exception.^173, 174^ Consistent with this, evidence suggests a role of gonadal hormones in suicidal behavior. Decreased testosterone levels have been correlated with increased propensity for depression^175, 176, 177^ and suicide attempts,^178, 179, 180, 181^ whereas increased testosterone has been associated with increased aggression in men.^182^ Moreover, it has been shown that women are more likely to attempt suicide when estrogen and progesterone levels are low. Suicide attempts made under these conditions have greater severity.^183^ Furthermore, perimenopausal women have higher suicidal ideation rates compared with women in pre- or postmenopause stages or compared with men, independent of mood disorders.^184^ In contrast to women, increased progesterone levels in adolescent men have been associated with increased suicidal thoughts and behavior.^185^ Although clinical studies have been designed to unravel the exact role of the menstrual cycle and female hormones in relation to suicidal behavior, results have been controversial with some studies reporting no relationship.^186, 187^ However, other studies report a higher risk of suicide during the premenstrual phase,^188, 189, 190^ menstruation,^190, 191, 192, 193^ or during the first and fourth weeks of the menstrual cycle.^194^ However, the majority of these studies based their assessment of menstrual cycle phases on interview methods, rather than an objective measurement. Moreover, postmortem studies investigating the role of menstrual cycle phase by assessing endometrial histology cannot avoid bias, as some suicides have been reported as non-suicidal fatalities.^195^ Therefore, animal models of suicide will be helpful to study the exact role of gonadal hormones in suicidal behavior, or endophenotypes associated with such behaviors.

Rodent models have been used over the years in an effort to thoroughly understand the hormonal influences on suicide-related endophenotypes, including impulsivity, anhedonia, and aggression. For instance, administration of progesterone, or both estrogen and progesterone, decreases impulsive behavior in ovariectomized rats.^196^ Moreover, in male rats, gonadectomy decreased impulsive action in the five-choice serial reaction time task compared with intact rats, suggesting that low testosterone levels might be associated with decreased impulsivity.^197^ In contrast, ovariectomy increased impulsive action in females in the same task.^197^ In addition, testosterone, dihydrotestosterone, estradiol, and progesterone administration have been shown to manifest antidepressant effects in rodents, as assessed by the forced-swim test.^198, 199, 200^ Consistently, testosterone or estradiol replacement prevented anhedonia phenotypes in gonadectomized male rats.^201^ On the other hand, increased testosterone levels are positively correlated with increased aggression in male rodents^202, 203^ and administration of medroxyprogesterone, a form of progesterone, increases male–male aggression and decreases male–female aggression in monkeys.^204^ Interestingly, the gene encoding for estrogen receptor alpha (*ER_α_*) is required for high levels of aggression in male mice ,^205^ whereas mice lacking the estrogen receptor beta (*ER_β_*) gene are characterized by a hyperaggressive phenotype,^206^ further demonstrating a role of gonadal hormones in endophenotypes associated with suicidal behavior. Overall, the findings from both clinical and preclinical studies suggest that circulating levels of gonadal hormones may affect suicide-related endophenotypes and the vulnerability to suicide.

The effects of gonadal hormones on suicide-related endophenotypes may be integrated with those of 5-HT dysregulation, discussed above, as testosterone increases 5-HT transporter mRNA expression and binding in rats and humans.^207, 208^ In addition, testosterone increases the neuronal firing rates of serotonergic neurons in the dorsal raphe nucleus in rats.^209^ Furthermore, the effects of testosterone on 5-HT transporter mRNA, binding, and serotonergic neuronal firing are thought to be dependent on aromatization of testosterone to 17β-estradiol.^208, 209^

## Personality and cognitive traits

### Aggression

A widely replicated association between suicidal behavior and quantitative behavioral measures is with indicators of aggression, also clinically observed as ‘impulsive aggression' (see the following refs 210, 211). These measures satisfy criteria for endophenotypes being heritable, associated with suicidal behavior, state independent, and co-segregated with suicidal behavior in families. In particular, retrospective studies suggest that suicide attempts are linked with aggression.^212, 213, 214, 215^ These associations also seem to hold for death by suicide. For example, Brent *et al*.,^216^ using the psychological autopsy method, reported that adolescents who died by suicide have higher levels of lifetime aggression than the healthy controls. These associations are independent of psychopathology.^217^ Prospective studies confirmed an association between higher levels of aggression and suicidal behavior.^218^ Moreover, the familial transmission of suicidal behavior appears to be mediated by transmission of impulsive aggression.^211, 219^ Family studies provide additional support for the association of aggression and suicide. For example, first-degree relatives of individuals with suicide attempts or ideation have a significantly higher history or levels of aggression.^219, 220, 221, 222^ Consistently, higher ratings of aggression are documented in families with a higher incidence of suicide attempts.^17^ However, the impact of witnessing aggression or abuse or being a victim of aggression or abuse cannot be disregarded. The use of animal model approaches presents opportunities to study the role of suicide risk factors in mediating aggression and impulsivity.

### Impulsivity

Impulsivity is another personality trait that has been strongly associated with suicidal behavior, and meets specific endophenotype criteria. Several retrospective studies have shown that suicide attempters and completers score higher on measures of impulsivity than controls.^212, 214, 223, 224, 225, 226^ Retrospective studies assessing suicide attempters have found that quantitative laboratory measures indicate higher levels of impulsivity.^227, 228^ In addition to retrospective studies examining impulsivity and suicidal behavior, several prospective studies have been carried out that look at baseline traits in relation to future suicidal behavior. For example, a study by Caspi *et al*.^229^ found that a group of toddlers that had initially been labeled as impulsive showed a higher subsequent frequency of suicidal behavior later in life. These data indicate that impulsivity is likely to be a stable trait over time and that this characteristic at a young age predisposes to suicidal behavior later in life. A separate study followed patients with mood disorders for 2 years. Subjects who attempted suicide during the follow-up had higher scores on a self-report of impulsivity at the beginning of the study.^218^ In terms of genetic epidemiology, among suicidal probands, those who had siblings who also attempted suicide showed the highest levels of impulsivity (as well as impulsive aggression).^230^ These data indicate that suicidal behavior and impulsivity may load together in families, thus further strengthening the impulsivity–suicidal behavior relationship.^219, 222^ What remains unknown is the relative contribution of kinship versus household exposure to environmental effects. Although almost impossible to tease apart in clinical samples, except in very special circumstances (for example, studying suicidal behavior in Old Order Amish), efficiently investigating ‘nature' versus ‘nurture' in relationship to impulsive aggression strongly supports a need for the use of animal models.

Although impulsivity is not a unitary construct, but rather a collection of behaviors that likely have separate neurobiological substrates, common definitions of impulsivity often include the themes of decreased inhibitory control, inability to delay reward, and impaired decision-making due to lack of consideration of possible outcomes. There are rodent behavioral tasks capable of measuring these behaviors, which include delay discounting (cognitive impulsivity) and the five-choice serial reaction time task (motor impulsivity).^231^

### Impaired decision-making

Impaired decision-making has repeatedly been identified as a trait of those with a history of depression and suicidal behavior in comparison to patients with histories of depression and no suicidal behavior, or healthy controls.^232, 233, 234, 235^ Importantly, this finding in euthymic patients, independent of comorbid psychiatric disorders, suggests that impaired decision-making represents a potential endophenotype of suicide vulnerability. It has been hypothesized that poor decision-making would influence the choice of immediately rewarding outcomes (for example, cessation of psychological pain) through long-term maladaptive solutions (suicidal act), when experiencing distressing events. Decreased activation of the lateral OFC during risky versus safe choices was associated with poorer decision-making in suicide attempters,^233^ suggesting that the decreased ability of these patients to correctly learn to recognize long-term risk in uncertain situations may represent key processes in the vulnerability to suicidal behavior.^236^ This bolsters the neuroanatomical hypothesis proposed by the results of postmortem and imaging studies,^164, 237^ suggesting that impaired serotonergic input to the prefrontal cortex may modulate the vulnerability to suicidal behavior.^18^

One method of identifying and measuring decision-making deficits in humans is using the Iowa Gambling Task (IGT) that simulates real life decision-making, and is subserved by the OFC.^238, 239, 240, 241^ At a behavioral level, it is necessary to deconstruct this task into component parts, that is, cognitive, motivational, and response processes, to identify which components of the IGT show impairment associated with suicidal behavior, as performed previously with other disorders.^242^ Decisions that involve uncertainty, options with multiple features, and changes over time place particularly high demands on cognitive control.^243^ However, poor decision-making and impaired cognitive control were not strongly inter-correlated but, rather, poor decision-making and cognitive control impairments appeared to independently, yet synergistically, contribute to suicidal behavior.^244^ Thus, the two processes may be supported by two independent pathways, the first cognitive control/frontoparietal pathway involves an inability to find and implement alternative solutions in a crisis. The second ‘value/paralimbic' pathway, involves a low threshold for suicidal acts, and a disregard of consequences and deterrents.^245^

Decision-making deficits are a potential endophenotype of suicidal behavior^31, 246^ since several genetic variants previously related to suicidal behavior^247^ and interacting with early maltreatment^248^ modulate the learning process necessary for choosing the advantageous options in the task. It is hypothesized that genetic variations alter efficiency of the neurotransmission in key brain regions involved in the learning process necessary for advantageous decision-making in uncertain conditions, and consequently increase the risk of suicidal behavior.

As decision-making is a complex process, further translational studies are needed to explore network and connectivity characteristics of identified variations in neural substrates as well as the molecular underpinnings of these variations. Modeling decision-making in animal models has strong potential to address these questions. Indeed, rodent versions of the IGT (r-IGT) exhibit good face and construct validities.^249, 250^ 5-HT transporter levels modulate long-term decision-making in this task in the rat (as shown in humans).^251^ r-IGT impairment is associated with a decrease in 5-HIAA levels in the OFC in a rat chronic pain model.^252^ The links observed in such models between 5-HT metabolism, OFC function, and decision-making are of relevance, as individual deficiencies in these three parameters have been implicated in suicide vulnerability. Reviewing studies based on the r-IGT, van den Bos *et al*.^253^ recently proposed that two different prefrontal-striatal networks were involved in task-progression in the r-IGT: an emotional/limbic system involved in assessing and anticipating the value of different options in the early stages of the task (learning task contingencies), and a cognitive control system involved in instrumental goal-directed behavior in later stages (behavior directed toward long-term options, reinforcement/punishment). Thus, animal models have the advantage to define complex neurocognitive and anatomical processes involved in decision-making and to examine developmental and environmental influences on decision-making.^254^

In addition to studies of neurobiology, the r-IGT could become a useful tool to study the biological basis of the decision-making performance-altering effects of pharmacological treatments. For instance, rats' ability to perform in the r-IGT is sensitive to drugs that modulate 5-HT and dopamine levels.^255^

There are growing interests in using noninvasive modulation techniques to clarify the neurobiology of suicidal behavior. The stimulation of the prefrontal cortex (ventromedial or dorsolateral) using repetitive transcranial magnetic stimulation, as well as transcranial direct current stimulation, in healthy volunteers induce changes in decision-making,^256^ generates emotional signals,^257, 258, 259, 260^ and modulates healthy subjects' ability to detect emotional cues.^261, 262^ Before these treatment modalities can be applied as a therapeutic tool for decision-making deficits in general, and suicidal behavior specifically, there is a clear need for a better understanding of their mode of action through the combined use of interventional clinical research and animal models. Models of transcranial direct current stimulation and repetitive transcranial magnetic stimulation in small animals have been adapted and tested in a wide range of behavioral paradigms^263, 264^ as they provide a powerful tool to identify the mechanisms by which transcranial direct current stimulation and repetitive transcranial magnetic stimulation modulate neural networks and the optimal parameters of stimulation, which could lead to more effective clinical interventions.

## Environmental risk factors

### Allergens and allergy

Data from large epidemiological studies have confirmed previously reported associations between asthma and suicide, and have identified, for the first time, significant links between allergic rhinitis and suicide after accounting for history of asthma.^265^ Considering that the massive peak of atmospheric pollen during spring overlaps with highly replicated seasonal peaks of suicide, investigators have hypothesized that inflammatory signals induced by pollen in the airways of sensitive subjects can induce suicidal behavior.^266^

In the first study on high aeroallergen exposure and suicide, a significant association between relative rates of suicide in women and tree-pollen levels were identified among those living in the continental United States.^267^ Although this finding was not replicated by the same group in a subsequent study in the United States,^268^ the underlying hypothesis was later confirmed in a large population study in Denmark.^269^ Furthermore, increased gene expression for allergy-related cytokines (such as IL-4 and IL-13) was found in regions of the prefrontal cortex previously implicated in suicide, specifically in the OFC.^134^ Very similar cytokine signals were identified in the prefrontal cortex of rodents sensitized and exposed to allergens.^270^ These rodents manifested increased anxiety behavior^270^ and alterations in social interactions,^270^ both considered risk-elevating factors for suicidal behavior.^271, 272^ A recent systematic review confirms an association between allergic disease and suicidal behavior, in particular with suicide mortality.^273^ Although evidence related to non-fatal suicidal behavior was considered not as strong as for fatalities, a recent study replicated the relationship between tree-pollen counts and fatal suicidal behavior in women, and additionally reported a positive relationship between grass pollen and attempted suicide for both genders.^274^ It is possible that the released mediators of allergic inflammation, rather than just symptoms of allergy, may increase risk of suicidal behavior, as in a pharmacoecological study, intranasal corticosteroids (known to reduce multiple mediators of inflammation) have been associated with lower suicide rates.^275^ In contrast, new generation antihistamines that primarily act via blockade of histamine and do not reduce the production of many other mediators of allergy, despite a similar level of improvement in allergy symptoms as intranasal corticosteroids, were associated with slightly elevated suicide rates.^275^ This could be the consequence of antihistamines not opposing mediators that reach the brain to the same degree, or possibly, a result of a direct pharmacological effect. This comparative paradigm could be used further in testing these alternative interventions for endophenotypes of suicide in animal models.

### Microbial pathogens

Pathogens are targets for and triggers of immune activation. As a consequence, they can activate pathways leading to alterations of emotion and behavior as described above (see ‘Inflammation' section). However, neurotropic pathogens might also have specific effects on neurons, or other cell types, in the brain.

A recent large Danish population study estimated that hospitalization for infection predicts subsequent suicide attempt with a population-attributable risk of infection accounting for 10.1% of suicide.^276^ Although this study identified nonspecific associations with various pathogens, suggesting perhaps a common denominator such as an immune mediation, other specific associations have also been identified. For instance, influenza B (not A) seropositivity has been associated with history of suicide attempts.^277^ Now replicated by multiple groups, a significant link was reported between *Toxoplasma gondii,* a highly prevalent^278^ latency establishing neurotropic intracellular parasite, and suicidal behavior across diagnostic categories.^277, 279, 280, 281, 282, 283, 284^ Importantly, in several studies, the links between *T. gondii* infection and suicidal behavior have been robust to adjusting for indicators of mental illness.^280, 285, 286^ A recent cohort study identified a statistical trend of an association between *T. gondii* and subsequent suicide attempt,^287^ consistent with a previous large cohort study in Danish mothers that found a predictive association between *T. gondii* infection and subsequent suicide attempts.^279^ Nevertheless, causality and the direction of causality have not been demonstrated.

The associations between chronic infection with *T. gondii* and suicide endophenotypes of aggression and impulsivity traits (gender- and age-specific) have been reported in both psychiatrically healthy individuals^283^ and psychiatric patients with clinically relevant impulsive aggression, that is, patients with intermittent explosive disorder.^288^ In rodents, latent *T. gondii* infection reduces and even reverses innate fear of cat odor and other stimuli that precede predation.^289^ Morphologically, latent infection with *T. gondii* induces dendritic retraction in the basolateral amygdala (a finding rendering neurophysiological support for reduced fear and anxiety-like behavior previously reported in infected rodents).^290^ The reported increased impulsivity in males, particularly younger males with *T. gondii* seropositivity,^283^ is paralleled by the recent identification of impulsive choices in rodents who had chronic infection with *T. gondii*,^291^ a model that allows pharmacological probing of impulsivity attributed to chronic infection with the parasite.

### Hypoxia

Recent *in vivo* neuroimaging studies found that healthy residents living at moderate altitude (1500 m, Salt Lake City, UT, USA) exhibit significantly higher whole-brain pH, lower inorganic phosphate^292^ and lower creatine levels in the anterior forebrain^293^ than age- and gender-matched healthy residents at sea level (Belmont, MA, USA or Charleston, SC, USA). Both inorganic phosphate and creatine have important roles in regulating energy metabolism, and low brain levels of these markers in healthy people at altitude signify low mitochondrial function, implying an altitude-related increase in vulnerability to major depressive disorder (MDD), bipolar disorder, and other psychiatric conditions linked to brain hypometabolism.^294^

Chronic exposure to hypoxia via living at a high altitude (hypobaric hypoxia) or with chronic hypoxic diseases, has recently been linked to significantly higher rates of MDD and suicide.^295^ Living at a high altitude appears to be an independent risk factor for suicide. In the United States,^294, 296, 297, 298^ South Korea,^298^ Austria,^299^ and Spain,^300^ although all-cause mortality rates tend to decrease with altitude,^297^ MDD rates increase with altitude of residence,^301, 302^ and suicidal ideation was found to be higher in MDD patients at high altitude versus those at sea level.^303^ Similarly, the odds ratios of both MDD and suicidal behavior are increased to ⩾100% for people with chronic hypoxic diseases, such as chronic obstructive pulmonary disease,^304^ asthma, and cardiovascular disease, versus in those with chronic diseases without hypoxia (diabetes, osteoarthritis), or those without a chronic disease.^305, 306, 307, 308, 309, 310^ Furthermore, these odds ratios increase with the severity of hypoxic disease^311, 312^ and with the current versus past status of hypoxic disease.^313, 314^ Chronic hypoxia was therefore proposed to worsen MDD severity and increase rates of treatment-resistant depression.

A novel translational animal model has been recently characterized to explore the etiology of high rates of MDD and treatment-resistant depression rates at altitude.^315^ The rats were housed for a week at altitude simulations of sea level, 3000 m or 6000 m or at local conditions of 1500 m (Salt Lake City, UT, USA) and then tested for depression-like behavior in the forced-swim test. Increasing the altitude of housing for a week, by itself, was found to incrementally increase depression-like behavior,^315^ thus providing construct validity for hypoxia-related depression. In rodent models, hypoxia lowers brain 5-HT levels,^316, 317^ and leads to brain hypometabolism via a deficit in the bioenergetic marker creatine.^318, 319^ Brain deficits in 5-HT levels and impaired mitochondrial function are also linked to MDD,^320, 321^ thus demonstrating face validity for this model.

As mortality by suicide is highly linked to unresolved depression,^322^ the impact of hypobaric hypoxia on antidepressant efficacy was also examined. Selective serotonin reuptake inhibitors, the most widely prescribed antidepressants, have been shown to lose antidepressant efficacy in other animal models of low brain 5-HT.^323, 324^ In this model, housing of rats at altitudes of 1400 m or 3000 feet for a week abolished the anti-immobility effects of the selective serotonin reuptake inhibitors (fluoxetine, paroxetine and escitalopram) in the forced-swim test,^325^ but not of the tricyclic antidepressant, desipramine.

Hypoxia has been shown to alter brain neurochemistry and physiology towards expression of several endophenotypes of suicidal behavior mentioned in this review. The cell culture and animal studies show that hypoxia can alter the synthesis and metabolism of neurotransmitters, including 5-HT, dopamine, norepinephrine, γ-aminobutyric acid (GABA), and glutamate.^326^ Of particular importance, rat brain 5-HT levels drop with hypoxia,^316, 317^ and low brain 5-HT in humans is linked to greater depression, impulsivity, risky behavior, and aggression, each of which is connected to suicidal behavior (see ‘Serotonin system'). Furthermore, hypoxia increases mitochondrial-mediated inflammation,^327^ inflammatory cytokines and pro-apoptotic markers in key cortical regions,^328^ increases HPA axis stimulation^329^ and lowers brain cellular metabolic function.^318, 319, 330^

Both demographic and human neuroimaging studies suggest that chronic hypoxia may function in myriad ways to alter brain chemistry, physiology and behavior. Studies related to the pathophysiology of chronic hypoxia-related brain dysfunction connected to suicide and specifically of corrective therapeutics are likely to derive a major benefit from the use of animal models, with the potential of rapid translation into clinical studies and better preventative and therapeutic options for suicidal behavior.

### Traumatic brain injury

Those with a history of traumatic brain injury (TBI), including all severity levels, are at significantly increased risk of suicidal ideation,^331, 332^ suicide attempts^331, 332^ and dying by suicide.^333, 334, 335, 336^ Simpson and Tate^337^ reported that, in outpatients with TBI, 23% had suicidal ideation within the previous 7 days, independent of time post-injury. The same authors reported that 17.4% of outpatients with TBI had attempted suicide over a mean period of 5 years. Meanwhile, Teasdale and Engberg^334^ reported a fourfold higher risk of death by suicide in those with TBI. A recent longitudinal cohort study over a 20-year period revealed that even a diagnosis of concussion results in increased suicide risk, which was estimated to be three times the population norm.^338^

TBI is associated with more extreme scores on measures of personality and cognitive traits associated with suicidality, including an increased sense of hopelessness, aggression, impulsivity, and impaired decision-making. Simpson and Tate^337^ reported a high level of hopelessness in those with TBI, with 34.9% scoring at moderate-to-severe levels of hopelessness, while hopelessness was a strong predictor of suicidal ideation. Aggression is a common consequence of TBI, with prevalence estimates of post-TBI aggression ranging from 11 to 34%,^339, 340, 341^ which may present as either verbal or physical aggression. TBI results in increased impulsivity,^342, 343, 344, 345^ in association with impaired decision-making and poor judgment.^343^ Based on a survey of four dimensions of impulsivity (urgency, lack of premeditation, lack of perseverance, and sensation seeking) in subjects with TBI, TBI resulted in increases in multiple dimensions of impulsivity, including urgency, lack of premeditation, and lack of perseverance.^342^ Finally, studies have described impaired decision-making or judgment abilities in those with TBI,^343^ including impaired decision-making in the IGT,^346^ in association with abnormalities in brain circuits implicated in decision-making.^347^

Neuroinflammation secondary to TBI is a cardinal feature of TBI and may serve as an endophenotype that can be evaluated in animal models. Mounting evidence indicates that neuroinflammatory processes start immediately after the initial TBI, and persist and worsen with time, contributing to the neurodegenerative process. Both microglia and astrocytes have important roles in neuroinflammation. Microglia are rapidly activated following TBI, which is manifested in: (i) morphological changes such as hypertrophy and de-ramification of processes; (ii) enhanced migratory and phagocytic activities; and (iii) production of inflammatory mediators including leukotrienes, cytokines and chemokines.^348, 349^ Astrocytes become reactive following TBI, which are manifested in: (i) increased proliferation (astrogliosis); (ii) migration towards injured tissues to form a glial scar; (iii) hypertrophy with increased expression of intermediate filaments (for example, glial fibrillary acidic protein); and (iv) the production of inflammatory mediators and growth factors that act via autocrine and paracrine signaling.^348, 349^ Although the initial activation of microglia^350^ and astrocytes is critical to wound healing, prolonged activation can lead to a self-perpetuating cycle of damaging events that drive the pathogenic processes underlying neurodegeneration. Evidence suggests that the elevation of markers such as IL-6 and C-reactive protein within the first 24 h post-trauma leads to worse outcomes for TBI.^349, 351, 352^ The increase in cerebral inflammatory response, including microglial and astroglial activation, is prolonged, lasting months or years, and is believed to contribute to the evolving symptomatology and pathology, thereby highlighting inflammation as a potential treatment target long after the acute trauma.^349, 353, 354^ For example, certain anti-inflammatory pharmacological interventions including statins, cyclosporine A, and glucocorticoids, have been investigated in animals and are undergoing clinical trials in TBI.^355^ Molecular consequences of TBI, such as cytokine activation and elevated levels of kynurenines^356, 357^ have been recently linked with suicidal behavior (see 'inflammation' section). It is thus plausible that neuroinflammation following TBI, at least in part, biologically mediates the link between TBI and suicidal behavior, and that anti-inflammatory interventions now studied could reduce the excess burden of suicidal behavior in TBI. Hypotheses exploring the links between TBI, inflammation, and suicidal thoughts and behavior, could be studied in animal models of TBI, focusing on endophenotypes of suicidal behavior and potential immune-mediating mechanisms.

In summary, in humans, TBI elevates the expression of certain endophenotypes of suicidal behavior, and increases risk of suicidal ideation, suicidal attempts, and suicide. Of interest to the thesis of this review, is whether or not TBI in animal models also increases either the molecular endophenotypes or cognitive and behavioral traits related to suicidal behavior. Neuroinflammation is a well-documented consequence of TBI in rodents,^358^ and negative behavioral outcomes of TBI, such as increased anxiety-related behaviors, can be prevented or reversed by drugs that inhibit microglial activation.^359, 360^ Cope *et al*.^361^ found that a controlled impact to the frontal cortex resulted in anhedonia, as measured in the sucrose preference test in rats. Consistently, studies in both mice^362, 363, 364^ and rats^365, 366^ have found that TBI also increases measures of behavioral despair as measured in the forced-swim test. Negative findings have also been reported,^367, 368, 369, 370^ but TBI paradigms in rodents frequently involve more limited and localized injury, and, based on studies in humans,^347^ behavioral effects would only be anticipated when relevant circuits are impacted by the injury. The tests of motor activity are consistent with increased impulsivity in rodents following TBI (for review, see ref. 371). In line with these findings, TBI in rats impairs impulse control in the five-choice serial reaction time task in association with neuroinflammation.^372^ Based on their findings, the authors concluded that neuroinflammation may represent a treatment target for impulse control impairments following injury. Few studies have evaluated the effects of TBI on aggressive behaviors in rodents (for review, see ref. 371), and this remains an important area for future studies.

## Medications that modify risk for suicidal behavior

Drugs with recognized antisuicidal or pro-suicidal effects may provide insight (and pharmacological predictive value) to the validity of potential animal models of suicidal behavior. Below, we discuss evidence for lithium, clozapine, antidepressants, and ketamine. Though not included, we also note that there is evidence that electroconvulsive therapy may be effective in the reduction of suicidality, and that electroconvulsive shock protocols exist for modeling the procedure in rodents.^373, 374^

### Lithium

Lithium remains one of the most valuable treatments for bipolar disorder and evidence indicates that lithium therapy reduces suicide risk.^375, 376, 377, 378^ Although lithium has not yet earned a United States FDA indication for the reduction of suicidal behavior, most of the randomized^379^ and nonrandomized^380, 381^ studies that have examined lithium's relationship to suicidal behavior are consistent with lithium reducing the risk of suicidal behavior in both bipolar and unipolar depression.

Anticonvulsants are popular clinical alternatives to lithium for the treatment of bipolar disorder. In this context, while controversial, the United States FDA has issued warnings regarding possible pro-suicidal effects of anticonvulsants.^382^ Thus, these potential alternative treatments for treating mood disorders do not have the evidence to support a beneficial role in reducing suicide/suicide behavior risks that lithium does, further increasing the need to understand whether, and what, is unique about lithium's impact on the neurobiological underpinnings of suicidal behavior. Furthermore, while lithium has some antidepressant actions, antidepressants such as selective serotonin reuptake inhibitors, at least in some populations,^383^ have been associated with an increased risk of attempting suicide in some studies. Other observations suggest that the reduction in suicide and suicidal behavior risks associated with lithium treatment does not appear to result solely from improvement of the underlying mood disorder.^384^

Furthermore, the association between lithium in the water supply across geographic regions and suicide rates has been studied. Most,^299, 385, 386, 387, 388, 389, 390^ but not all,^391^ of these studies associate higher levels of lithium in drinking water with lower rates of suicide. One enigma posed by these data is that the levels of lithium in the drinking supply are calculated to result in daily doses of only approximately 1%, or in some cases, close to 0.1% of typical clinical doses.^392^ The results have been inconsistent regarding possible greater effects in either males^385^ or females.^386^ Lithium's mechanism of action relevant to these findings remains to be elucidated, especially considering that most preclinical studies have been performed with lithium doses that achieve blood levels observed with the treatment of mood disorders.^393^ It is also worth considering—and could be experimentally addressed in animal models—that the alternative interpretation of these data is that low lithium levels are a risk factor for suicide, rather than higher lithium levels being a protective factor. Of relevance, continuous exposure to similar concentrations of lithium to those found in drinking water, have been shown to reduce mortality in a *C. elegans* model.^394^ Investigation is needed to explore the possibility that lithium may interact with a unique molecular target to reduce suicide risk, both at levels proven efficacious for the treatment of mood disorders, and at lower doses that may have efficacy for reducing suicidal behaviors.

There are a number of important opportunities for animal models and animal research to advance the understanding of lithium or lithium-like molecules related to their potential for reducing the risk of suicide. Mood stabilizers often affect common molecular targets^395^ but they do not all decrease suicide risk; therefore, future research may identify the neurobiological changes that lithium brings about, but which other mood stabilizers do not.^395, 396^ Lithium may exert its antisuicidal actions by modifying aggressive and impulsive behaviors. This hypothesis is supported by numerous double blind, placebo-controlled studies suggesting an anti-aggressive effect of lithium across various populations.^397, 398, 399, 400^ The results of randomized, placebo-controlled studies also suggest that lithium decreases human impulsivity. However, the evidence for such an effect is not as strong as for aggression, and concurrent diagnoses of pathological gambling and bipolar disorder in some studies make the interpretation complicated.^398, 401, 402^ Lithium's attenuation of suicide endophenotypes, including aggression and impulsivity, can also be readily modeled in rodent models using behavioral tests.^403, 404, 405^ Direct comparison with other mood stabilizers, such as valproate, may be beneficial. For example, while valproate can attenuate aggression in some animal tests, it is not as effective as lithium in reducing impulsivity in certain paradigms.^404, 406, 407^ Uncovering the mechanisms leading to these differences might help to pinpoint the specific antisuicidal actions of lithium. One strategy has been to investigate the molecular actions of lithium at the genetic and neurobiological levels using various inbred and transgenic mouse models.^393, 408, 409, 410^ Nonhuman animal studies allow the consideration of time course and dose–response experiments.^411, 412^ Data indicating that environmental exposure to lithium (based on concentrations in the water supply) is inversely proportional to suicide rates, at least on a population basis, suggest the utility of determining whether there is an unusual dose response curve for lithium's impact on aggressive behaviors or other models of suicide endophenotypes in nonhuman animals. As potential molecular actions of lithium are identified, it would be useful to examine whether dose–response relationships are evident concerning the impact on these targets, which might explain the apparent effectiveness at particularly low doses as those measured in the water supply. Lithium was recently associated with improved decision-making in bipolar patients.^413^ The molecular dissection of the effects of lithium on impulsivity (potentially through modulating 5-HT neurotransmission) may help to identify potential mechanisms underlying the antisuicidal effect of lithium, and provide new molecular targets for medications that reduce the risk of suicide.

Another role for animal models may exist in helping facilitate the search for ‘lithium mimetic' drugs with decreased potential for adverse events. The need to address suicidal behavior risk is often acute, while the time frame for experiencing any adverse effects from lithium treatment is often over years to decades. Adverse effects of lithium on end organs such as the kidneys, thyroid, and parathyroid glands typically take years to even begin to manifest. For instance, in some cases, such as renal insufficiency in men under the age of 60 years, individuals receiving one or more lithium prescriptions, and control individuals, have renal function that appears indistinguishable for more than 20 years,^414^ yet, the methodology used to examine such long time frames has limitations. An ideal treatment for suicidal behavior should lack any possible concern about gradually accruing end organ damage. This is an opportunity where investigations in animals would help advance the search for a compound that would convey some or all of the benefit of lithium on suicidal behavior (or at least the most appropriate endophenotypes), while not sharing lithium's adverse effects. Last, the possibility of ‘rebound' mood episodes or suicidal behavior after lithium discontinuation is a possible limitation to its use as a short-term suicide prevention strategy.^415^ Animal models may allow investigation of whether rebound aggressiveness, impulsivity, or other behaviors occur after sudden withdrawal of lithium, but not of other medications. Thus, it can be appreciated that animal research with lithium has great potential to clarify its mechanisms of actions and potentially allow the development of lithium-like, yet safer, medications, establish dose response and identify safety-based approaches to mitigate side effects to be further tested clinically in humans.

### Clozapine

Clozapine is the only medication with United States FDA approval for use to reduce suicidal behavior. The indication is specifically for reducing suicidal behavior in patients with schizophrenia. In a large randomized controlled trial (InterSEPT study, *n*=980), clozapine was associated with a reduction in suicidal behavior and rescue hospitalizations compared with olanzapine, although not suicide deaths (overall, eight suicides were observed).^416^ Clozapine has effects on a large number of neurotransmitter receptors, including multiple dopaminergic, serotonergic, muscarinic, adrenergic, and histaminergic receptor subtypes.^417^ It is not known why clozapine exerts these antisuicidal behavior effects, or for certain the degree to which they may be related to its impact on treatment of refractory schizophrenia. It is hypothesized that clozapine's nonselectivity for neurotransmitter receptors may underlie its distinct efficacy.^418^ Clozapine's distinct reduction in mood symptoms in patients with schizophrenia may be important to its effects of reducing suicidal behaviors.^419^ The impact of these or other candidate mechanisms on suicidal behavior has yet to be elucidated. Studies with clozapine could provide valuable clues to the neurobiology of suicide in animal models and may include an assessment of common mechanisms of action between lithium and clozapine. For example, similar to anti-aggression-like effects of lithium, clozapine has also been demonstrated to reduce aggressive behaviors in a chronically stressed mouse model.^420^ As is also the case for lithium, research concerning how to prevent some of clozapine's sizable side-effect burden may help make it more desirable for wider use, or identify a clozapine-like molecule that preserves the efficacy but not the toxicity of clozapine.

### Antidepressants

Due to space considerations, we will avoid a detailed discussion of antidepressants. However, antidepressants are the only medication known to have a highly age-dependent effect on suicidal behavior. Randomized trial meta-analyses have suggested that antidepressants are associated with an increased risk of suicidality among patients of the youngest age as noted by the FDA black box warning,^421, 422^ and are associated with reduced risk of suicidality among the oldest patients.^421^ As such, antidepressants provide an opportunity to probe the age-related changes that occur in the brain paralleling this unusual apparent reversal of the effects of antidepressants on suicidality. Extending research of antidepressants of suicide endophenotypes in developmental (young and old) animal studies could help clarify the neurobiological systems important to suicidal behavior.^371^

### Ketamine

Ketamine has been shown to have fast-acting effects to decrease suicidal thoughts.^423, 424, 425^ Ketamine's fast action makes it a particularly valuable research tool to develop biomarkers of response and to more precisely understand the neurobiology of antidepressant and antisuicidal response.^426, 427^ Indeed, recent studies are beginning to uncover neural circuitry involved in ketamine's rapid effects to reduce suicidal thoughts.^428^ In contrast to lithium and clozapine, for which the only available evidence concerning suicide/suicidal behavior risk relates to patients taking the medication for days, weeks or months, ketamine can rapidly reduce suicidal ideation within a time frame that can be measured in minutes and hours rather than weeks.^424, 429, 430, 431^ Moreover, it has been demonstrated that ketamine might have anti-aggressive properties depending on the experimental model used,^432^ which requires further investigation. Thus, it remains to be confirmed whether ketamine has similar actions as lithium does on impulsivity and aggression relevant to suicidal behavior. Considering the rapid-acting nature of ketamine on suicidality in general, and suicidal thoughts, in particular, ketamine may be impacting suicidality in a manner distinct from those of lithium or clozapine. The recent finding that ketamine's *in vivo* conversion to a hydroxynorketamine metabolite is necessary and sufficient for its antidepressant actions, without its side effects, in mice presents an additional opportunity to understand the mechanism whereby ketamine rapidly reduces suicidal thoughts.^433^

### Drugs that may increase risk of suicide

Medications ranging from asthma to acne treatments have received warnings about increased suicidal ideation or behavior. Therefore, screening and/or understanding why these effects are observed using animal models may be useful. For instance, rimonabant, an inverse agonist for the cannabinoid receptor CB1, was initially marketed as an anti-obesity drug.^434^ However, after some time of use in clinical practice, it was removed from the market due to concerns about increased suicide risk.^435^ Later rodent studies demonstrated that chronic treatment with this drug increased immobility time in the forced-swim test and increased anhedonia as measured by sucrose preference.^436^ Rimonabant has also been shown to decrease 5-HT levels in the frontal cortex, and to adversely affect neurogenesis and immune function.^436^ Of note, effects of rimonabant on impulsivity measures in animals have been equivocal. Although the drug increased impulsivity in the delay-discounting paradigm, it decreased some impulsive behaviors on the five-choice serial reaction task, which measure different subdomains of impulsivity.^437, 438, 439^

Isotretinoin, used to treat severe cases of acne since the 1980s, has been associated with an increase of suicidal behavior in vulnerable individuals.^440, 441, 442^ Studies in mice showed that chronic treatment with this drug increased immobility time in the forced-swim test and tail suspension test, suggesting increased behaviors or relevance to depression neurobiology.^443^ Surprisingly, given the potential pro-suicidal effects of the drug, chronic isotretinoin treatment reduced aggression in the resident-intruder test.^444^

Despite the mixed findings with the two drugs discussed here, pro-suicidal properties of drugs may be partially quantifiable by focusing on particular endophenotypes of suicide in animal models, therefore providing detailed information about molecular pathways related to changes in suicide risk. A systematic study of these drugs and others that may modify risk may explain the conflicting associations of increased suicidal risk suggested in the past for drugs such as montelukast,^445^ varenicline^446^ and interferon-α.^447, 448, 449^

## Conclusions

In clinical research on suicide, understanding individual vulnerabilities, resiliencies, deterrents, precipitating and perpetuating factors for each patient and the vast variety of personal circumstances leading to their suicidal behavior is a challenging task. The effects of undergoing current and previous drug treatments, and compensatory mechanisms in response to comorbid substance abuse, psychiatric or medical conditions and treatments only further complicate matters. Based on cumulative results, a general model has been proposed postulating that vulnerability to suicidal behavior is mediated in part by an important underlying genetic predisposition interacting with environmental and probable epigenetic factors throughout the lifespan. This combination of risk factors then modifies the function of neuronal circuits involved in behavioral modulation, thus rendering an individual more likely to engage in a suicidal act^18, 450^ (Figure 1). However, the factors that lead to suicide are tremendously complex, multifaceted, and heterogeneous. Using animal model approaches that allow us to experimentally study the neurobiology underlying suicide endophenotypes is a promising and much needed layer in suicide research. The planned expanded use of research domain criteria, versus categorical symptom checklist-based diagnosis, presents a further opportunity to support such an endeavor.^452^ Although our review is broad, it is by no means comprehensive. We did not include several translatable domains that are associated with increased suicide risk and potential targets for interventions. These include sleep impairment, substance use disorders, and mixed mood states. Furthermore, the review did not include complex interactive models of suicidal behavior, the modeling interactive effects of risk factors, and the domain of helplessness that has human and animal correlates.^453^ Although highly valuable for their translational relevance, knowledge is lacking to construct these multivariable models before answering the simpler questions proposed in this review.

Animal models for studying complex behaviors have been very successful in guiding research in humans, which has led to important discoveries of clinical relevance. In addition, animal studies have been crucial in validating observations from human samples, where studies are limited by ethical considerations and where access to brain tissue is restricted to rare postmortem samples. Furthermore, confounding factors, such as comorbidities, different ages, and different life experiences, are unavoidable in human samples. Although informative of changes associated with patient histories, psychiatric illnesses and suicidal behavior, the use of postmortem brain samples precludes the study of molecular changes occurring at the onset of suicidality. The discrete and dynamic changes occurring at the onset of disease may be the point at which clinical intervention would be the most beneficial. A better understanding of such changes, along with technological or diagnostic advances to detect such changes, would allow for faster and more effective treatments.

Considering endophenotypes as potential targets for new treatments, tested first in animal models and then in humans, may enable us to circumvent certain obstacles in human studies of suicidal behavior, represented by ethical concerns, high comorbidity and confounding factors, as well as issues with obtaining adequate sample sizes.^454^ Evaluation of new treatments for suicidal ideation and behavior could be based on the involvement of identified cognitive and emotional brain circuits, related to dysfunction of subregions of the cortex and other regions occurring in the pathophysiology of suicidal behavior.

Ultimately, animal models may provide opportunities to directly test the functional role of genetic variants associated with suicide-related endophenotypes, in particular, impulsivity, aggression, and decision-making impairments. With the advent of CRISPR/Cas9 and other genome-editing technologies, it is now more straightforward to directly model human genetic variants in mice.^455^ Such models provide an invaluable opportunity to also investigate gene by environment interactions and identify sensitive developmental periods, two factors that may contribute to the lack of replication of many genetic associations. Such approaches may help clarify the literature regarding genetic variants implicated in suicide^456^ and provide insight into the circuit-based mechanisms that contribute to different suicide-implicated endophenotypes.

Overall, research aimed at elucidating the neurobiology of suicidal behavior in animal models that allows uncovering and engaging novel treatment targets and discoveries, and early screening of treatments to prevent and reduce suicidal behavior, could be utilized to a much greater degree in suicide research. This will take time to establish, but, in our view, will ultimately succeed in the longer run—if integrated with efforts at multiple levels, such as macroepidemiological, clinical (in particular interventional), postmortem—to reduce suicide mortality, a public health priority that has proven, so far, resilient to therapeutic interventions and societal investments implemented to date.
